# Editorial: Neurotrophins Biodelivery to CNS: Innovative Approaches for Disease-Modifying Therapy

**DOI:** 10.3389/fnins.2022.916563

**Published:** 2022-05-10

**Authors:** Viviana Triaca, Bruno Pietro Imbimbo, Robert Nisticò

**Affiliations:** ^1^Institute of Biochemistry and Cell Biology, National Research Council, Rome, Italy; ^2^Research and Development, Chiesi Farmaceutici, Parma, Italy; ^3^Department of Biology, School of Pharmacy, University of Tor Vergata, Rome, Italy; ^4^Laboratory of Pharmacology of Synaptic Plasticity, Fondazione EBRI Rita Levi Montalcini, Rome, Italy

**Keywords:** neurotrophins, neurodegenerative diseases, blood-brain barrier, focused ultrasound, intranasal route

Neurodegenerative diseases (NDs) are expected by 2050 to rise to 300% worldwide. Patients affected by Alzheimer's Disease (AD), Parkinson's Disease (PD), Huntington Disease (HD), and Amyotrophic Lateral Sclerosis (ALS) experience gradual and progressive neurons loss and neuronal function deterioration, which frequently result in cognitive dysfunctions and severe impairment of activities of daily living. Due to the unknown etiology of these neurological disorders and the difficulty of an early diagnosis, we are currently experiencing a lack of effective and disease-modifying treatments (Erkkinen et al., 2018).

Neurotrophins (NTs) belong to a class of growth factors which regulate survival, development and function of neurons through the Trk-p75 NTR receptors system (Chao and Hempstead, 1995). Secreted neurotrophic factors act by preventing the target neurons from initiating programmed cell death, thus allowing the neurons to survive (Patapoutian and Reichardt, 2001). NTs also induce differentiation of progenitor cells to form neurons. Although the vast majority of neurons in the mammalian brain are formed prenatally, parts of the adult brain (for example the dentate gyrus of the hippocampus and subventricular zone) retain the ability to grow new neurons from neural stem cells, a process known as adult neurogenesis. NTs are able to help to stimulate and control neurogenesis (Park and Poo, 2013).

The progressive deficient activity of neurotrophic factors like nerve growth factor (NGF), brain derived neurotrophic factor (BDNF) and glial cell-derived neurotrophic factor (GDNF) in different neurodegenerative diseases has prompted intensive research to identify neurotrophic-based disease modifying therapies (Allen et al., 2013). BDNF has been reported to affect cognitive activity via its specific receptor tyrosin kinase B (TrkB) in a variety of neurological and psychiatric disorders (Lu et al., 2014), including PD (Stefani et al.) and chronic social stress (Cui et al.).

NTs and their pro-forms, namely the proneurotrophins (ProNTs), exert opposite effects on adult neurons, with the former generally being neuroprotective and the latter promoting a degenerative pattern (Patapoutian and Reichardt, 2001; Lu et al., 2005). In line, the inhibition of the BDNF precursor ProBDNF attenuates inflammation and encephalopathy in an animal paradigm of CNS sepsis (Jiang et al.).

NGF plays a major role in the maintenance of cholinergic baso-cortical and baso-hippocampal circuits involved in memory and higher cognition, and is considered a critical molecule for integrity and function of cholinergic neurons during development and adulthood. Numerous studies have also shown that NGF contributes to the survival and regeneration of neurons during aging and in age-related diseases such as AD (Cuello et al., 2019). Changes in neurotrophic signaling pathways are involved in the aging process and contribute to cholinergic and cognitive decline observed in AD (Volosin et al., 2006). Based on these biological properties, NGF has been proposed to restore cognition, halt neuroinflammation, and revert degeneration not only in AD, but also in Down's syndrome, neurotrauma, as well as in eye diseases, like macular degeneration (Alastra et al.; Capsoni and Cattaneo; Manni et al.; Amadoro et al.).

A mutated form of NGF has been proposed as a valuable neurotrophic substitute of the wild-type molecule without its pain-related side effects (Capsoni and Cattaneo).

Nonetheless, NGF- and BDNF-based clinical trials for AD and ALS, respectively, have failed probably due to poor brain penetration and insufficient target engagement. So far, the poor pharmacokinetic properties of neurotrophins render their use for the treatment of CNS disorders limited. To overcome this issue, in the last 15 years, the nasal route of administration for neurotrophins and peptidomimetics has been widely attempted in animal and human settings with promising preliminary results (Tessarollo and Yanpallewar; Alastra et al.; Manni et al.).

Several small neurotrophic agents that efficiently cross the blood-brain barrier (BBB) have been designed and tested in preclinical and clinical research. Recently, a European consortium, the first of its kind, has been intended to foster the study of neurotrophin mimetics in neuroinflammation and neurodegenerative diseases [EuroNeurotrophins, 2018-2022, H2020-MSCA-ITN-2017 (ETN), doi 10.3030/765704]. Among these molecules, the most promising are specific blockers of the p75NTR receptors. Additional compounds designed to activate Trk receptors signaling have been demonstrated to improve synaptic loss and memory deficits (Longo and Massa, 2013). Also, the TrkB.T1 truncated adult form of TrkB has gained attention for the distinct BDNF actions in brain physiopathology, pinpointing both epigenetic and splicing-related regulatory control of the neurotrophic pathway even beyond the nervous system (Tessarollo and Yanpallewar).

In the last decade, neuroscientists resorted to the nose-to-brain route of drugs delivery as non-invasive approach to bypass the BBB. Different forms of nanocarriers, including liposomes, nanopolymers, and emulsions have been attempted to transport drugs directly into the brain. However, and despite the potential of nasal delivery, optimal pharmacokinetics and target engagement are still waiting for significant optimization (Barbato et al.).

The transcranial high intensity focused ultrasound (FUS) is a ground-breaking technology for ablative surgery. FUS technology has been approved by FDA for the treatment of essential tremors and Parkinson's disease-related tremors. At lower intensity, FUS can stimulate or inhibit neural activity with potential applications in clinical practice for epileptic seizures and psychiatric disorders, as well as for chronic pain management by temporarily blocking nerves. Recently, preclinical studies demonstrated how low intensity FUS can transiently and finely regulate the BBB opening, thereby facilitating the brain penetrance of drugs especially when combined with intranasal drug delivery (Barbato et al.; Ji et al., 2019). FUS technology could therefore represent a game changer in the long-running battle against several neurodegenerative diseases with an emphasis on treating AD and PD.

## Conclusions

Compelling evidence accumulated over the last two decades strongly pinpoint neurotrophins as neuroprotective and possibly disease-modifying treatments for the treatment of neurodegenerative diseases. Despite this growing hope, neurotrophins-based therapies are still suffering from major biological limitations including poor pharmacokinetic properties and very low penetration rates through the BBB. The exploitation of a new and adaptable technology like FUS, which allows non-invasive, efficient, and focused drug delivery to the brain, would possibly open a new era in the treatment of developmental and age-related deadly illnesses of the CNS.

There is promise for brain therapy. It will be up to the scientific community and drug developers to take a chance on it.

## Author Contributions

All authors equally contributed to the editorial conceptualization and writing, approved the submitted version.

## Conflict of Interest

BI is an employee at Chiesi Farmaceutici. He is listed among the inventors of a number of Chiesi Farmaceutici's patents of anti-Alzheimer drugs. The remaining authors declare that the research was conducted in the absence of any commercial or financial relationships that could be construed as a potential conflict of interest.

## Publisher's Note

All claims expressed in this article are solely those of the authors and do not necessarily represent those of their affiliated organizations, or those of the publisher, the editors and the reviewers. Any product that may be evaluated in this article, or claim that may be made by its manufacturer, is not guaranteed or endorsed by the publisher.
